# Trisomy 8 clonal expansion during disease progression and azacitidine resistance in VEXAS syndrome: a case report

**DOI:** 10.3389/fimmu.2026.1841156

**Published:** 2026-06-10

**Authors:** Keijiro Sato, Yoshitaka Zaimoku, Yui Kano, Miku Kobayashi, Shintaro Kazama, Takahiro Morikawa, Hiroko Kazumoto, Wataru Ishii, Toshimitsu Ueki, Masahiko Sumi, Hikaru Kobayashi, Yasuhito Nannya, Seishi Ogawa, Naoaki Ichikawa

**Affiliations:** 1 Department of Hematology, Japanese Red Cross Nagano Hospital, Nagano, Japan; 2 Department of Hematology, Kanazawa University Hospital, Kanazawa, Japan; 3 Innovate Clinical Research Center, Kanazawa University Hospital, Kanazawa, Japan; 4 Department of Clinical Laboratory Science, Division of Health Sciences, Graduate School of Medical Science, Kanazawa University, Kanazawa, Japan; 5 Department of Rheumatology, Japanese Red Cross Nagano Hospital, Nagano, Japan; 6 Division of Hematopoietic Disease Control, Institute of Medical Science, The University of Tokyo, Tokyo, Japan; 7 Department of Pathology and Tumor Biology, Kyoto University, Kyoto, Japan; 8 Institute for the Advanced Study of Human Biology (WPI-ASHBi), Kyoto University, Kyoto, Japan; 9 Department of Innovative Medicine, Faculty of Medicine, Kindai University, Osakasayama, Japan; 10 Department of Medical Oncology, Japanese Red Cross Nagano Hospital, Nagano, Japan

**Keywords:** azacitidine resistance, cord blood transplantation, myelodysplastic syndrome, trisomy 8, VEXAS syndrome

## Abstract

**Background:**

Vacuoles, E1 enzyme, X-linked, autoinflammatory, somatic (VEXAS) syndrome is an adult-onset autoinflammatory disorder driven by clonal hematopoiesis with somatic *UBA1* mutations. Azacitidine can reduce the mutant clonal burden and improve inflammatory manifestations. However, the clonal basis of treatment resistance remains poorly understood.

**Case presentation:**

A 61-year-old man presented with predominant gastrointestinal symptoms, macrocytic anemia, thrombocytopenia, and systemic inflammation. A bone marrow examination revealed cytoplasmic vacuoles in myeloid precursors and trisomy 8. Retrospective genetic testing identified a somatic *UBA1* p.Met41Val variant, thus establishing the diagnosis of VEXAS syndrome in this patient. At the time of referral and during immunosuppressive therapy with prednisolone and cyclosporine, the *UBA1* variant allele frequency was consistently higher than the trisomy 8 burden, although relapse coincided with an expansion of trisomy 8. Azacitidine reduced the clonal burdens of both the *UBA1* mutation and trisomy 8. However, both persisted at similar levels, and after 21 cycles, the disease progressed to myelodysplastic syndrome with increased blasts-2, accompanied by concordant re-expansion of *UBA1*-mutated and trisomy 8 clones. These findings suggest that a trisomy 8 subclone likely emerged within a *UBA1*-mutated founder clone and expanded during treatment, whereas the disomy 8 fraction of the *UBA1*-mutated clone was preferentially eliminated by azacitidine. Urgent cord blood transplantation resulted in sustained clinical and molecular remission.

**Conclusion:**

This case suggests that trisomy 8 may contribute to disease progression and azacitidine resistance in VEXAS syndrome.

## Introduction

Vacuoles, E1 enzyme, X-linked, autoinflammatory, somatic (VEXAS) syndrome is an adult-onset systemic autoinflammatory disorder caused by somatic mutations in the X-linked *UBA1* gene in hematopoietic stem cells (1–4). Most pathogenic variants involve methionine 41 and impair the ubiquitin-activating enzyme E1, resulting in dysregulated ubiquitination, endoplasmic reticulum stress, and excessive proinflammatory cytokine production (5–10). Emerging evidence suggests that this inflammatory state is not merely a downstream consequence of *UBA1* mutations but also contributes to the selective persistence and competitive advantage of *UBA1*-mutated clones (9).

Clinically, VEXAS syndrome is characterized by recurrent fever, neutrophilic dermatoses, pulmonary infiltrates, chondritis, venous thrombosis, arthritis, and hematologic abnormalities such as macrocytic anemia, thrombocytopenia, cytoplasmic vacuoles in myeloid precursors, and monoclonal gammopathy (1, 4, 7, 11, 12). Hematologic neoplasms, particularly myelodysplastic syndrome (MDS) and plasma cell neoplasms, are reported in approximately 25-50% of such patients (13–15).

The optimal treatment strategies remain uncertain. Immunosuppressive therapies may alleviate inflammatory manifestations; however, they generally do not eradicate *UBA1*-mutated clones or prevent hematologic progression (1, 11, 16–19). Allogeneic hematopoietic stem cell transplantation is potentially curative, but it carries considerable treatment-related risk (20–27). Azacitidine, a hypomethylating agent, has been reported to reduce the mutant clonal burden and improve systemic inflammation (28–33). A recent systematic review of 166 patients treated with hypomethylating agents reported inflammatory and hematologic responses in 59% and 74% of the cases, respectively, although the responses were not always durable (34). However, the clonal mechanisms underlying treatment resistance remain unclear.

We herein describe a patient with VEXAS syndrome in whom clinical relapse and hematologic progression paralleled the clonal expansion of trisomy 8.

## Case description

A 61-year-old Japanese man presented with a 1-month history of unexplained fever, right upper abdominal pain, myalgia, arthralgia, leukocytosis, and sore throat. Laboratory testing revealed neutrophilic leukocytosis (white blood cell count, 18.8 × 10^9^/L; neutrophils, 88%; lymphocytes, 9%; monocytes, 3%), macrocytic anemia (hemoglobin, 92 g/L; mean corpuscular volume, 107 fL), thrombocytopenia (platelet count, 117 × 10^9^/L). In addition, liver dysfunction (aspartate aminotransferase, 42 U/L; alanine aminotransferase, 43 U/L; alkaline phosphatase, 188 U/L; gamma-glutamyl transpeptidase, 132 U/L), elevated C-reactive protein (319 mg/L), and hyperferritinemia (1,821 µg/L) were observed. Rheumatoid factor and antinuclear antibodies were negative. He was provisionally diagnosed with atypical adult-onset Still’s disease. Prednisolone (1 mg/kg/day; 60 mg/day) led to a rapid clinical improvement. However, 5 months later, lower abdominal pain recurred during tapering to 17.5 mg/day, and he was referred to our institution.

**Figure 1:**
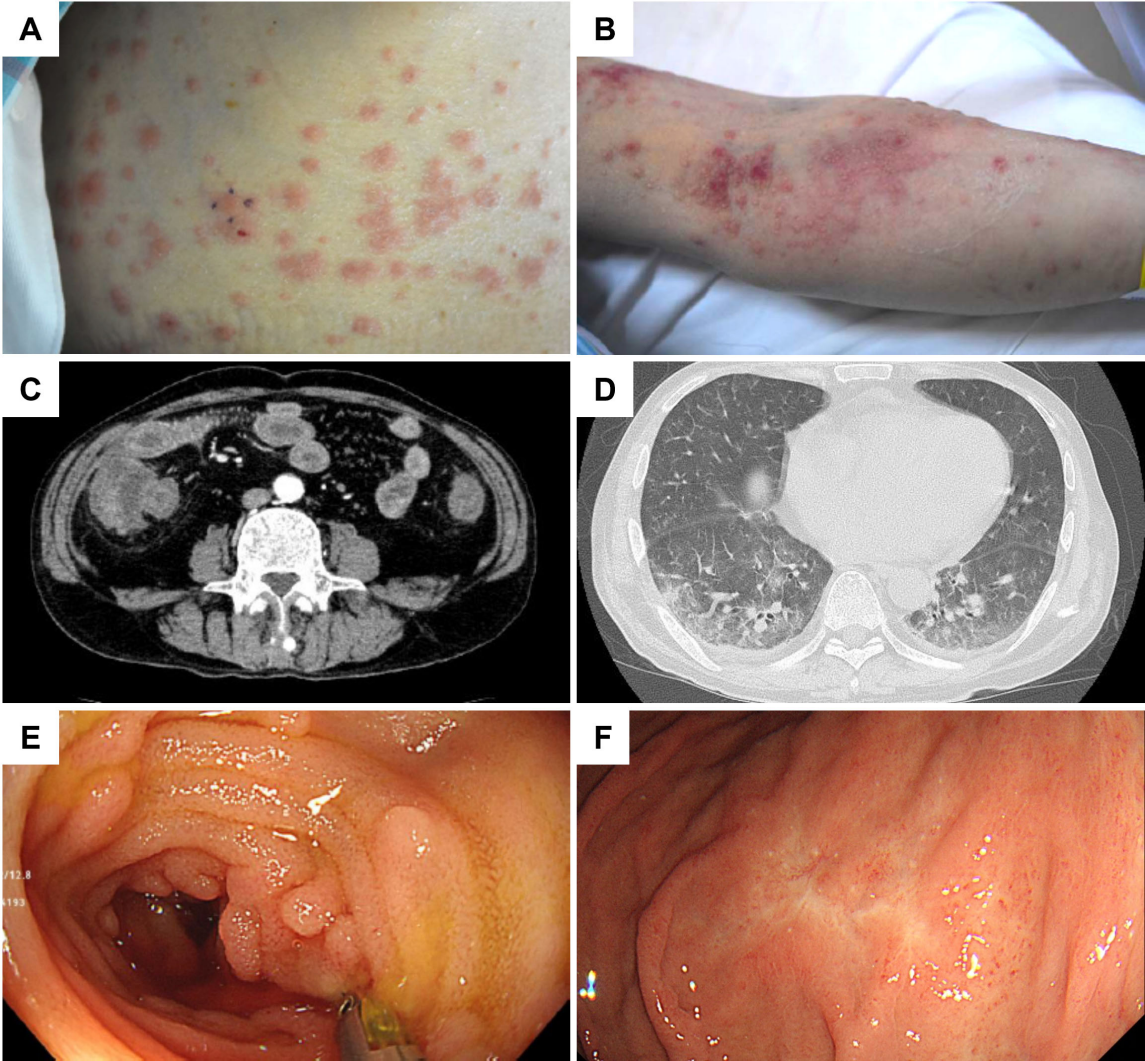
Clinical manifestations at referral. **(A)** Erythematous eruptions on the trunk. **(B)** Erythematous eruptions on the upper extremity. **(C)** Contrast-enhanced abdominal computed tomography showing diffuse wall thickening of the entire colon and distal small intestine. **(D)** Chest computed tomography showing bilateral ground-glass opacities. **(E)** Colonoscopy showing mucosal edema without ulceration in the terminal ileum. **(F)** Upper gastrointestinal endoscopy showing atrophic gastritis with multiple ulcer scars.

On referral, the patient had a persistent high-grade fever, lower abdominal pain, and profuse watery diarrhea (up to 20 episodes per day), accompanied by nausea. Erythematous to violaceous eruptions were present on the trunk and extremities (**Figures 1A, B**). There was no evidence of arthritis, polychondritis, oral or genital ulcers, or ocular involvement.

The laboratory findings were largely similar to those observed at the initial presentation, although the patient’s liver function had normalized. The white blood cell count was 16.4 × 10^9^/L (myelocytes, 2%; metamyelocytes, 3%; neutrophils, 85%; lymphocytes, 9%; monocytes, 1%). Hemoglobin was 82 g/L, mean corpuscular volume was 116 fL, platelet count was 98 × 10^9^/L, reticulocyte count was 48 × 10^9^/L, and the C-reactive protein level was 101 mg/L.

Contrast-enhanced computed tomography demonstrated diffuse wall thickening of the entire colon and distal small intestine (**Figure 1C**) and bilateral pulmonary ground-glass opacities (**Figure 1D**). Colonoscopy revealed mucosal edema without any ulceration in the terminal ileum (**Figure 1E**), whereas the colon was macroscopically unremarkable. Upper gastrointestinal endoscopy revealed atrophic gastritis with multiple ulcer scars (**Figure 1F**).

**Figure 2:**
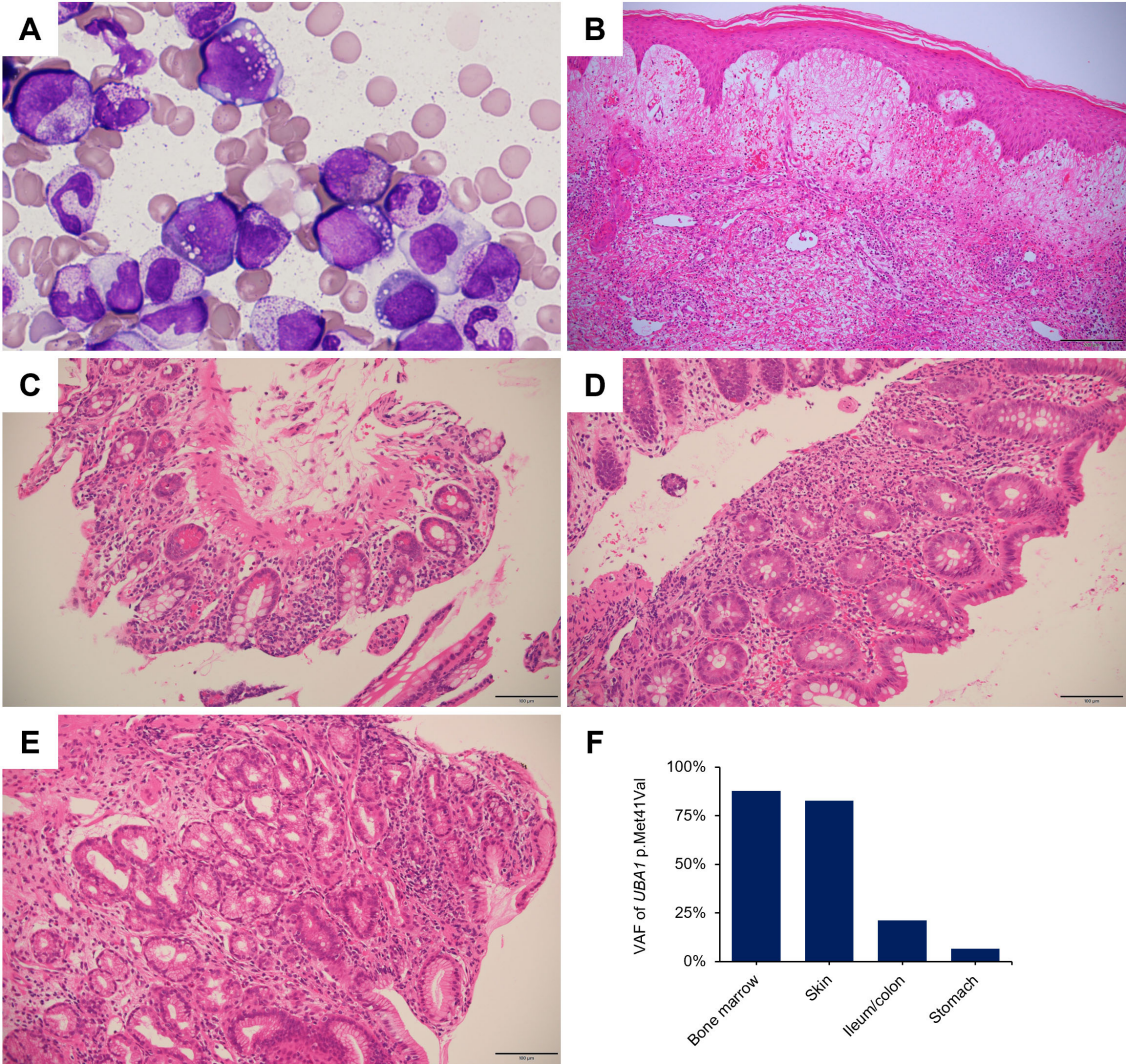
Histopathological findings and tissue distribution of the *UBA1* mutation. **(A)** A bone marrow aspirate smear showing cytoplasmic vacuoles in myeloid precursors. **(B)** A skin biopsy demonstrating marked papillary dermal edema with dense neutrophilic infiltration, consistent with Sweet syndrome (hematoxylin and eosin staining). **(C, D)** Biopsies from the ileum **(C)** and colon **(D)** showing mild chronic inflammatory infiltrates with interstitial edema and focal neutrophilic infiltration (hematoxylin and eosin staining). **(E)** A gastric biopsy demonstrating mild inflammatory cell infiltration in the lamina propria (hematoxylin and eosin staining). **(F)** Variant allele frequency (VAF) of the *UBA1* p.Met41Val mutation in bone marrow and tissue biopsy specimens as assessed by digital polymerase chain reaction.

Bone marrow aspirate showed myeloid hyperplasia with toxic granulation, erythroid hypoplasia, preserved megakaryopoiesis, and 0.6% blasts. Scattered cytoplasmic vacuoles were noted in myeloid and erythroid precursors without MDS-defining dysplasia (**Figure 2A**). G-banding identified trisomy 8 (47,XY, + 8) in 4 of 20 metaphases.

A skin biopsy showed marked papillary dermal edema and dense neutrophilic infiltration with eosinophils, consistent with Sweet syndrome (**Figure 2B**). Biopsies from the ileum and colon demonstrated mild chronic inflammatory infiltrates with interstitial edema and focal neutrophilic infiltration (**Figures 2C, D**). Gastric biopsy specimens showed mild inflammatory infiltrates (**Figure 2E**).

**Figure 3:**
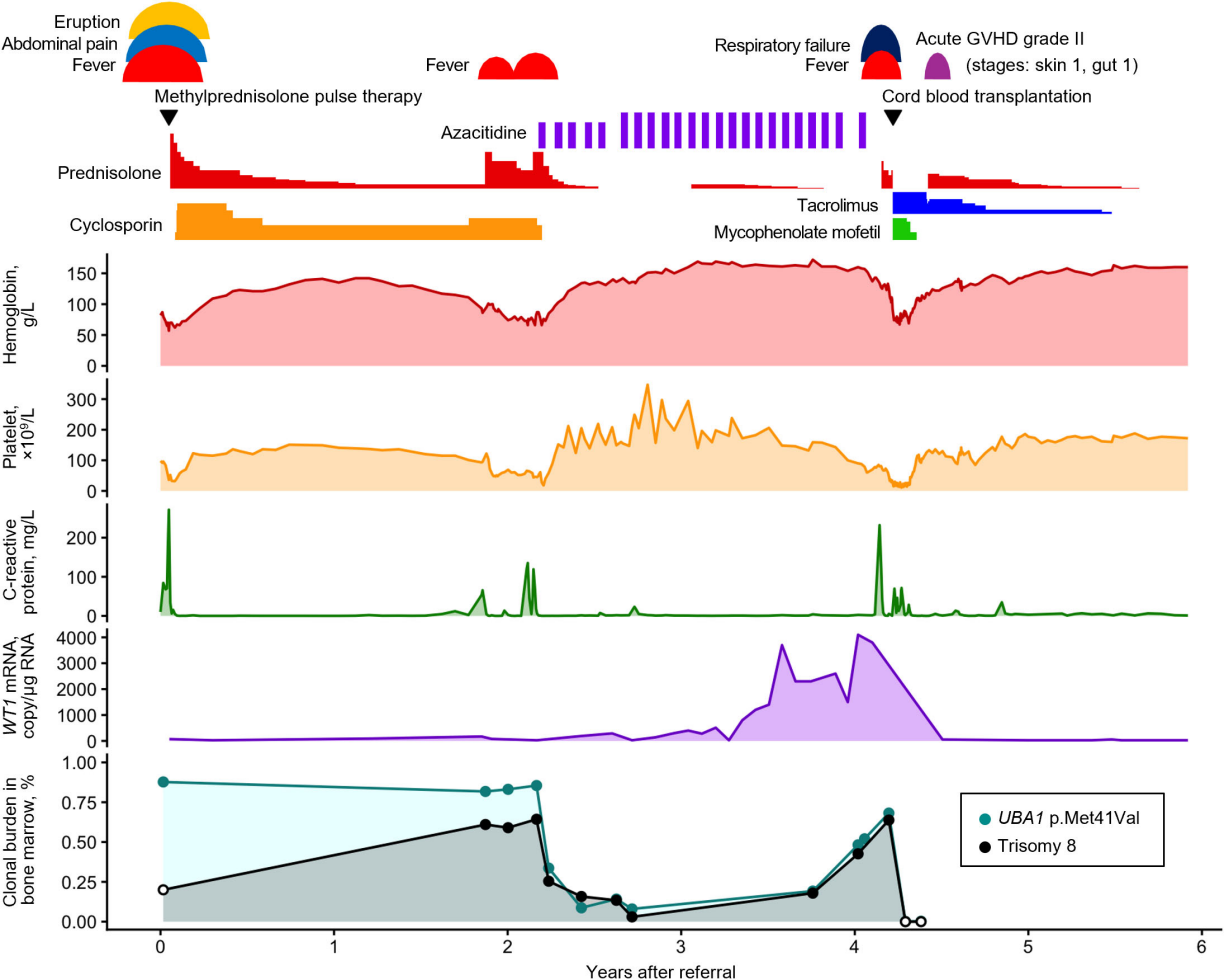
Clinical course and clonal dynamics. Upper panel: Treatment timeline and major clinical events, including methylprednisolone pulse therapy, prednisolone, cyclosporine, azacitidine (23 cycles), and cord blood transplantation. Middle panels: Serial changes in hemoglobin, the platelet count, serum C-reactive protein, and the *WT1* mRNA levels. Lower panel: The longitudinal clonal burden of the *UBA1* p.Met41Val mutation and trisomy 8 in bone marrow samples, assessed by digital polymerase chain reaction and fluorescence *in situ* hybridization, respectively. Open circles indicate trisomy 8 detected by G-banding.

As no unifying diagnosis had been established at that time, the patient received methylprednisolone pulse therapy followed by oral prednisolone (60 mg/day) and cyclosporine, with a marked improvement in the systemic and gastrointestinal symptoms, as well as cytopenias (**Figure 3**).

Approximately two years later, while prednisolone was gradually tapered to 5 mg/day, macrocytic anemia, thrombocytopenia, and pulmonary infiltrates recurred, whereas gastrointestinal and cutaneous manifestations remained in remission. A bone marrow examination showed persistent vacuolization without an increase in blasts, but trisomy 8 had expanded to 70% (14/20 metaphases) in G-banding and 64.3% (643/1000 uncultured interphase cells) in fluorescence *in situ* hybridization (FISH). Increasing the dose of prednisolone to 40 mg/day again resulted in a clinical improvement. However, inflammatory symptoms relapsed again during a subsequent taper of prednisolone (20 mg/day).

Based on the clonal expansion of trisomy 8 and the presence of apparently abnormal vacuolated bone marrow cells, despite the lack of any definitive morphological features, the patient was clinically diagnosed with MDS. Azacitidine (75 mg/m^2^/day for 5 days) was initiated, and cyclosporine was discontinued, leading to an improvement in cytopenias and pulmonary infiltrates. After three cycles, trisomy 8 decreased to 15.8% (158/1000 by FISH), and prednisolone was discontinued after four cycles. After five cycles, hematologic recovery became delayed, and a bone marrow examination revealed 5.0% blasts. Azacitidine administration was therefore extended to seven days per cycle, resulting in a reduction of the blasts to 2.4%, and the treatment was continued on this 7-day schedule.

Before the 22nd cycle, a bone marrow examination revealed 11.4% blasts and trisomy 8 in 42.6% (426/1000 by FISH), consistent with a progression to MDS with increased blasts-2. The peripheral blood *WT1* mRNA levels increased to 4,100 copies/μg RNA. Two additional cycles of azacitidine transiently controlled the blast counts. However, the patient subsequently developed high-grade fever and respiratory failure, requiring supplemental oxygen.

Because of azacitidine-refractory MDS with persistent systemic inflammation and the lack of a human leukocyte antigen-matched related donor, the patient underwent urgent cord blood transplantation after conditioning with fludarabine (180 mg/m^2^), busulfan (9.6 mg/kg), and melphalan (80 mg/m^2^), with tacrolimus and mycophenolate mofetil as graft-versus-host disease prophylaxis. The infused total nucleated cell count was 2.3 × 10^7^/kg and the CD34-positive cell count was 0.78 × 10^5^/kg. Neutrophil engraftment was achieved on day 22, and complete donor chimerism was confirmed on day 26 using sex chromosome FISH (recipient XY karyotype, 0/500; donor XX karyotype, 497/500; undetermined X karyotype, 3/500). The disappearance of trisomy 8 was demonstrated by G-banding. Although chromosome 8 FISH was not performed after transplantation to prioritize the chimerism analysis, the absence of a recipient karyotype indicates a high level of elimination. Grade II acute graft-versus-host disease (skin stage 1, gastrointestinal stage 1) developed on day 35 and was controlled with the reinstitution of prednisolone (20 mg/day). No chronic graft-versus-host disease or other major transplant-related complications occurred.

Three years after transplantation, the patient remained in complete remission without any immunosuppressive therapy. The patient reported satisfaction with the overall clinical outcome and has returned to normal daily life without significant limitations.

## Genetic analyses

To clarify the clonal basis of the patient’s multisystem inflammation, we performed target panel sequencing using the bone marrow aspirates obtained prior to cord blood transplantation, with a buccal swab as a germline control (Supplementary Methods), as previously described by us (35, 36). This analysis identified a somatic *UBA1* variant (p.Met41Val; c.121A>G) with a variant allele frequency (VAF) of 46.1%, thus establishing the diagnosis of VEXAS syndrome. However, the results became available after transplantation. In addition, low-level nonsynonymous variants in *TET2* (p.Pro1123Cysfs*6; VAF 3.2%; likely oncogenic) and *PHF6* (p.Lys241Glu; VAF 4.7%; variant of uncertain significance) were identified (**Supplementary Table 1**).

To evaluate clonal dynamics and composition, we retrospectively performed digital polymerase chain reaction (PCR) using paraffin-embedded bone marrow aspirate clot samples (Supplementary Methods), as described previously (27). The clonal burden was then compared with that of trisomy 8, assessed during the treatment course as part of routine clinical practice, using conventional metaphase G-banding and/or interphase FISH with a chromosome 8p11.1–q11.1 enumeration probe. Because the *UBA1* gene is located on the X chromosome in this male patient, the clonal burden was assumed to be equivalent to that of the VAF.

The *UBA1* mutation was detectable at the initial referral, with a VAF of 87.6%, clearly exceeding the clonal burden of trisomy 8 as detected by G-banding (20%, 4/20 metaphases), although FISH was not performed in the initial cytogenetic assessment. During prednisolone and cyclosporine therapy, the *UBA1* mutation burden remained consistently higher than the trisomy 8 burden; however, trisomy 8 expanded from 20% to 64–70% in parallel with treatment refractoriness (**Figure 3**, lower panel; **Supplementary Table 2**). After azacitidine initiation, both *UBA1*-mutated and trisomy 8 clones rapidly decreased, but persisted at similar levels, before subsequently undergoing parallel re-expansion. After transplantation, the *UBA1* mutation became almost undetectable in the recipient. Low-level variants in *TET2* and *PHF6* were also confirmed by digital PCR both at the diagnosis and before transplantation (**Supplementary Table 1**).

Digital PCR was also performed on biopsy specimens obtained during referral. The *UBA1* VAF was highest in the bone marrow (87.6%), followed by the skin (82.8%), ileum/colon (21.1%), and stomach (6.5%) (**Figure 2F**, **Supplementary Table 3**), supporting the widespread tissue infiltration by *UBA1*-mutated cells. The patient’s peripheral blood was not analyzed because of the retrospective nature of the study.

## Discussion

This case illustrates a plausible clonal model for disease evolution in VEXAS syndrome: a *UBA1* p.Met41Val-mutated founder clone gave rise to a trisomy 8 subclone that expanded during immunosuppressive treatment, persisted during azacitidine therapy, re-expanded at relapse with disease progression, and was ultimately eradicated by cord blood transplantation. In contrast, the disomy 8 compartment of the *UBA1*-mutated clone appeared to be preferentially eliminated by azacitidine. These observations suggest an association between trisomy 8 and disease progression, as well as resistance to azacitidine.

Although most patients with VEXAS have a normal karyotype, trisomy 8 has been reported in a small subset of cases. Available reports suggest that trisomy 8 is more frequently observed in relatively younger patients and in those with p.Met41Val mutations and MDS with increased blasts and it is associated with a higher likelihood of undergoing allogeneic transplantation (**Supplementary Table 4**) (20, 26, 27, 29, 37, 38). Of note, Gurnari et al. reported a case in which trisomy 8 emerged after azacitidine therapy prior to transplantation (26), and Diarra et al. described a case that required transplantation following refractoriness to azacitidine (20). Taken together, these findings are consistent with the potentially adverse clinical impact of trisomy 8 in VEXAS syndrome.

This interpretation is consistent with prior observations that isolated trisomy 8 in MDS and acute myeloid leukemia is associated with less favorable outcomes than normal cytogenetics, despite its conventional classification as an intermediate-risk abnormality (39–43).

Trisomy 8 has also been linked to autoinflammatory phenotypes, particularly Behcet’s disease-like syndromes with recurrent fever and gastrointestinal manifestations (44–49). Whether trisomy 8 directly amplifies inflammatory signaling or is selectively favored within an inflammatory marrow environment remains uncertain. Our findings primarily support the latter model, in which inflammation driven by the *UBA1*-mutated founder clone creates a permissive environment for the emergence of trisomy 8 as a fitter subclone under inflammatory and therapeutic pressure. Nevertheless, the copy-number gain involving *MYC* at chromosome 8q24.21 could potentially contribute to enhanced inflammatory signaling, as MYC activation has been implicated in the oncogenic stress-induced activation of innate immune pathways (50). Such a mechanism raises the possibility that trisomy 8 may further reinforce inflammatory signaling, thereby contributing to clonal persistence and treatment resistance.

Gastrointestinal involvement is increasingly recognized within the clinical spectrum of VEXAS syndrome, ranging from abdominal pain and chronic diarrhea to bleeding, perforation, and stenosis (11, 51, 52). In this patient, molecular analyses demonstrated *UBA1*-mutated cells in gastrointestinal biopsy specimens, thus supporting clonal involvement in gastrointestinal lesions.

The main strengths of this report are serial clonal analyses of both *UBA1* mutation and trisomy 8 using matched bone marrow aspirate samples, showing a distinction between the *UBA1*-mutated founder clone and the trisomy 8 subclone, and the integration of these findings with longitudinal clinical outcomes. Its limitations include the single-patient design, the lack of colony assays to confirm the clonal architecture, the absence of functional studies to prove a mechanistic role for trisomy 8 in azacitidine resistance, and the presence of minor *TET2* and *PHF6* clones, whose contributions cannot be excluded. In addition, the potential involvement of genomic alterations not covered by targeted sequencing, as well as epigenetic changes, cannot be ruled out. Nevertheless, this case underscores the importance of comprehensive cytogenetic evaluation and longitudinal clonal monitoring in VEXAS syndrome.

In conclusion, the detection or expansion of trisomy 8 in VEXAS syndrome may represent a high-risk feature that warrants close monitoring and timely consideration of definitive therapies, including hematopoietic stem cell transplantation. Further studies are therefore needed to validate the role of trisomy 8 in VEXAS syndrome.

## Data Availability

The original contributions presented in the study are included in the article/**Supplementary Material**. Further inquiries can be directed to the corresponding authors.
