# Supplementary material for: Trisomy 8 clonal expansion during disease progression and azacitidine resistance in VEXAS syndrome: a case report
Source: Front Immunol. 2026 Jun 10;17:1841156. doi: 10.3389/fimmu.2026.1841156 (PMC13290977; doi:10.3389/fimmu.2026.1841156)
Supplement: Supplementary file 1 [file DataSheet1.pdf]

Trisomy 8 clonal expansion during disease progression and azacitidine resistance in VEXAS syndrome: a case report

Supplementary Methods

Target panel sequencing

Genomic DNA (50 or 200 ng) obtained from bone marrow aspirate was enriched for target regions encompassing 454 genes by liquid-phase hybridization using a SureSelect custom kit (Agilent Technologies), according to the manufacturer's protocol optimized for automated sample processing, as previously described.[1,2] The purified libraries were subjected to high-throughput sequencing on a DNBSEQ-G400RS platform (MGI) using 150-bp paired-end reads. Variants were called and annotated using established methods as previously described.[1,2] Variants were interpreted according to the ClinGen/CGC/VICC criteria.[3]

The targeted 454 genes were as follows:

ABCB7, ABL1, ACIN1, ACSM2A, ADAMTS17, ADH5, ADHFE1, AKT1, AKT2, AKT3, ALAS2, ALDH1B1, ALK, AMIGO3, ANKRD26, APC, AR, ARHGEF10, ARID1A, ARID1B, ARID2, ASH1L, ASXL1, ASXL2, ATAD5, ATF7IP, ATG2B, ATM, ATR, ATRX, AURKA, BAALC, BAP1, BCAS1, BCL11A, BCL11B, BCL2, BCL2L1, BCOR, BCORL1, BLM, BOD1L1, BRAF, BRCA1, BRCA2, BRCC3, BRIP1, C15ORF65, C1QTNF3, CALR, CBFA2T3, CBFB, CBL, CBLB, CCDC7, CCL16, CCND1, CCND2, CCND3, CDAN1, CDC25C, CDH1, CDH23, CDK4, CDK6, CDKN1B, CDKN2A, CDKN2B, CEBPA, CECR1, CENPA, CHD3, CHD4, CHD8, CHD9, CHEK2, CLCN6, CMYA5, CREBBP, CSF1R, CSF2RA, CSF2RB, CSF3R, CSF3R,rs3917981, CSNK1A1, CSNK2B, CTC1, CTCF, CUX1, DAZAP1, DAZAP1,rs2271055, DCAF7, DCC, DCLRE1C, DDR2, DDX41, DHX15, DHX30, DICER1, DIS3, DKC1, DNAJC21, DNM2, DNMT3A, DOCK4, DOT1L, DST, DYNC2H1, E2F6, ECT2L, EED, EFL1,EFTUD1, EGFR, ELANE, ELF1, ELF4, EP300, EPOR, ERBB2, ERBB3, ERBB4, ERCC4, ERCC6L2, ERF, ERG, ESCO2, ETNK1, ETS1, ETS2, ETV6, EXOC3L1, EZH2, FANCA, FANCB, FANCC, FANCD2, FANCE, FANCF, FANCG, FANCI, FANCL, FANCM, FARSA, FBXW7, FGFR1, FGFR2, FGFR3, FGFR4, FLT3, G6PC3, GATA1, GATA2, GFI1, GFI1B, GIGYF1, GIGYF2, GJB3, GLRX5, GNA11, GNAQ, GNAS, GNB1, GNB2, GNE, GNL2, GNL3L, GPR171, GRIK2, GSE1, GSKIP, HAX1, HCFC1, HCFC2, HCN1, HDAC8, HIST1H3F, HIST1H3H, HLA-A, HLA-B, HLA-B,HLA-C, HLA-C, HLTF, HNRNPK, HOXC9, HRAS, HSPA9, HUNK, IDH1, IDH2, IDH3A, IDH3B, IFNA7, IGF1R, IGHMBP2, IKZF1, IKZF2, IKZF3, IRF1, IRF2, IRX1, ITGB1, JAK1, JAK2, JAK3, JARID2, KANSL1, KAT6A, KDM5A, KDM6A, KDR, KIF2B, KIR3DX1, KIT, KLC2, KLF1, KMT2A, KMT2C, KMT2D, KMT2E, KMT5B, KRAS, LCE3C, LIG4, LIN28A, LPA, LTN1, LUC7L2, MAD2L2, MAP2K1, MAP4K2, MBD4, MBNL1, MDM2, MECOM, MED1, MED12, MET, MGA, MLLT10, MN1, MNAT1, MPL, MRE11A, MTA2, MTOR, MYB, MYC, MYD88, MYH11, MYSM1, MZF1, NBEAL2, NCAPD2, NCOR1, NCOR2, NDUFB11, NEURL,NEURL1, NEURL1, NF1, NF2, NFE2, NFIA, NFIX, NHP2, NIPBL, NOL3, NOLC1, NOP10, NOTCH1, NOTCH2, NOTCH4, NPM1, NRAS, NRIP1,rs2229741, NSD1, NTRK1, NTRK2, NTRK3, NUP214, NUP98, NXF1, PAK1, PALB2, PARN, PCDHA1, PDE4DIP, PDGFRA, PDGFRB, PDS5B, PHF6, PHIP, PICALM, PIEZO1, PIGA, PIGT, PIK3C2A, PIK3C2B, PIK3CA, PIK3CG, PIM1, PIM2, PLCG1, PLXNA4, POM121L12, POSTN, PPM1D, PRF1, PRKDC,

PRMT1, PRPF8, PRR14L, PTCH1, PTEN, PTPN1, PTPN11, PTPRD, PUF60, PUS1, PXDNL, PYGO2, RAD21, RAD21L1, RAD51, RAD51C, RAF1, RB1, RBBP6, RET, RFWD3, RIT1, ROBO1, ROBO2, ROS1, RPL11, RPL15, RPL22, RPL26, RPL27, RPL31, RPL35A, RPL4, RPL5, RPS10, RPS15A, RPS17, RPS19, RPS24, RPS26, RPS27, RPS28, RPS29, RPS7, RRAS, RRAS2, rs11093377,HDAC8, rs20539,KDM6A, rs2238154,SH2B3, rs3930513,WT1, rs743589,MYB, rs916018,JAK3, rs999020,KIT, RTEL1, RUNX1T1, S100A8, S100A9, SAMD9, SAMD9L, SAMHD1, SBDS, SBF2, SDHA, SDPR, SETBP1, SETD1A, SETD1B, SETD2, SETX, SF1, SF3A1, SF3B1, SH2B3, SIDT2, SLC19A2, SLC24A1, SLC25A38, SLX4, SMARCA4, SMARCD3, SMC1A, SMC3, SMO, SNX13, SOS1, SPI1, SRCAP, SRP54, SRP72, SRSF2, SRSF7, STAG1, STAG2, STAG3, STAT3, STAT5B, STK11, SUZ12, SVEP1, SYK, TAF6, TCF4, TERF1, TERF2, TERT, TET2, TFIP11, THEMIS, TINF2, TLR2, TNRC18, TP53, TP53BP1, TPP1, TRNT1, TSC1, TSC2, TSR2, TYW5, U2AF1, U2AF2, UBA1, UBE2A, UBE2T, UBR4, UBTF, UROC1, USP9X, VEGFA, VHL, WAPAL, WDR92, WRAP53, WT1, XRCC2, YARS2, YLPM1, ZBTB33, ZBTB7A, ZEB2, ZFP36L2, ZFPM1, ZNF318, ZNF687, ZNF77, ZRSR2

Digital PCR

Digital PCR was performed to quantify the variant allele frequency (VAF) of individual variants in paraffin-embedded specimens. Genomic DNA was extracted from paraffin sections using a standard xylene-based protocol. Digital PCR was conducted using the QuantStudio Absolute Q digital PCR system and Absolute Q DNA Digital PCR Master Mix (Thermo Fisher Scientific), according to the manufacturer's instructions. Primer pairs and fluorescent-labeled probes (fluorescein for the variant allele and SUN for the wild-type allele) targeting the *UBA1* p.Met41Var variant was previously established,[4] whereas those for *TET2* and *PHF6* variants were newly designed and synthesized by Integrated DNA Technologies (Coralville, IA, USA).

Supplementary Tables

Supplementary Table 1. Non-*UBA1* variants in bone marrow.

Gene	Transcript cDNA change (protein change)	Classification (ClinGen/ CGC/VICC)	Time after referral	Variant allele frequency and digital PCR analysis images*
TET2	NM_001127208.3 c.3364_3365del (p.Pro1123Cysfs*6)	Likely oncogenic (OVS1 and OP4)	At referral	0.4% (digital PCR) 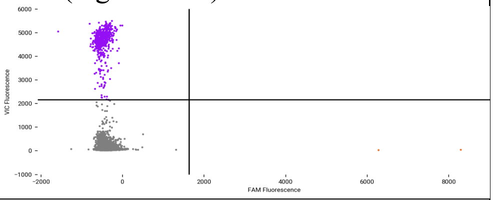
			48 months	3.2% (target sequencing)
			50 months	0.6% (digital PCR) 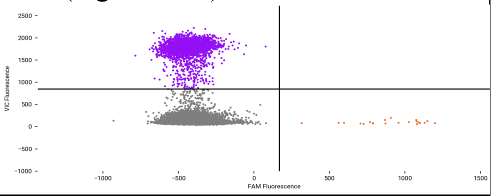
PHF6	NM_001015877.2 c.721A>G (p.Lys241Glu)	Variant of uncertain significance (OP4)	At referral	0.9% (digital PCR) 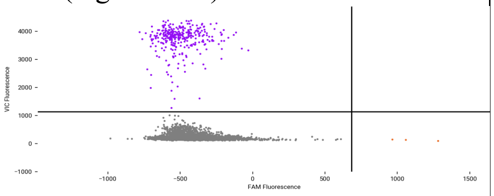
			48 months	4.7% (target sequencing)
			50 months	3.2% (digital PCR) 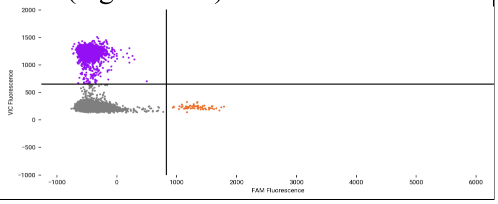

*Digital polymerase chain reaction (PCR) plots show variant-positive microwells (orange) and wild-type microwells (purple).
Abbreviations: NA = not assessed, OP4 = predicted functional impact, OVS1 = predicted loss-of-function; PCR = polymerase chain reaction.

Supplementary Table 2. Clonal dynamics of *UBA1* mutation and trisomy 8 in bone marrow.

Time after referral	Blast	Trisomy 8		Digital PCR results for *UBA1* p.Met41Val variant	
		G-band	FISH	VAF	Analysis images*
At referral	0.6%	20% [4/20]	NA	87.7%	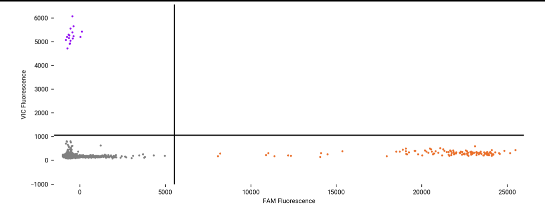
22 months During prednisolone + cyclosporine	1.6%	70% [14/20]	61.0% [610/1000]	81.8%	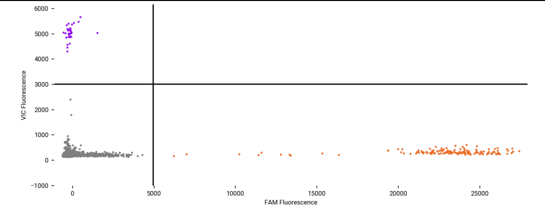
24 months During prednisolone + cyclosporine	1.4%	50% [10/20]	59.0% [590/1000]	83.1%	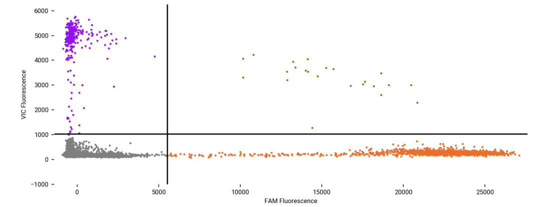
26 months During prednisolone + cyclosporine	0.4%	70% [14/20]	64.3% [643/1000]	85.5%	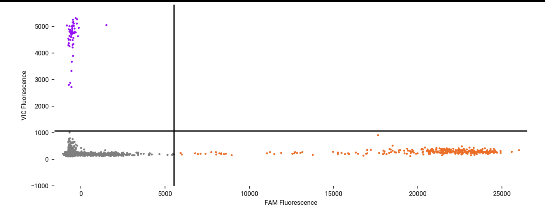
27 months After azacitidine #1	0.8%	35% [7/20]	25.4% [254/1000]	33.6%	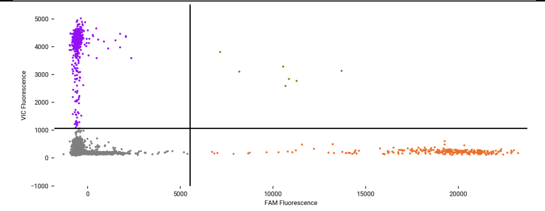
29 months After azacitidine #3	1.6%	5% [1/20]	15.8% [158/1000]	8.7%	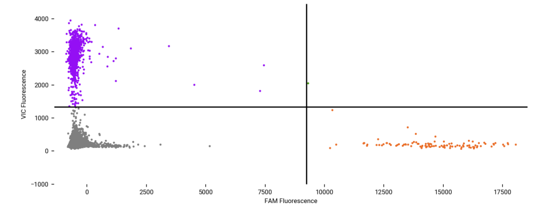
31 months After azacitidine #5	5.0%	15% [3/20]	13.4% [134/1000]	14.2%	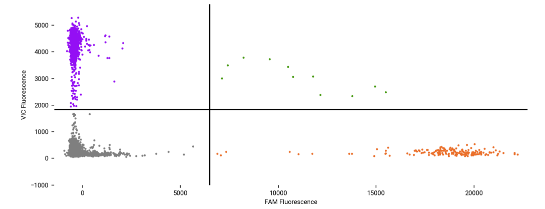

32 months After azacitidine #6	2.4%	5% [1/20]	3.0% [30/1000]	8.0%	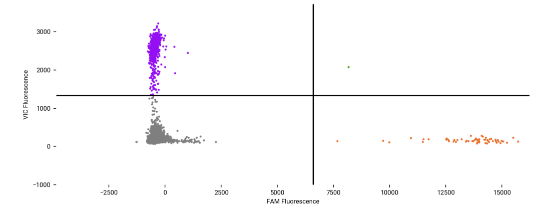
45 months After azacitidine #19	3.8%	15% [3/20]	17.9% [179/1000]	19.2%	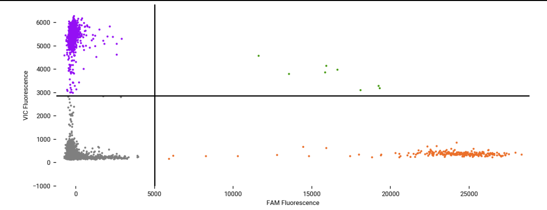
48 months After azacitidine #21	11.4%	75% [15/20]	42.6% [426/1000]	48.2%	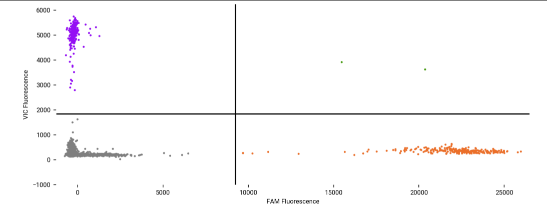
48 months After azacitidine #22	7.4%	NA	NA	52.0%	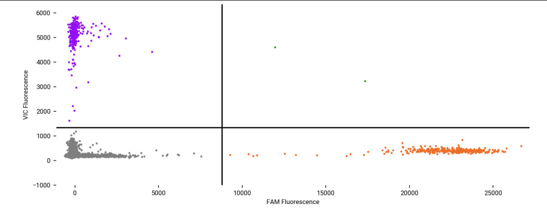
50 months After azacitidine #23 Before CBT	3.0%	80% [16/20]	63.8% [638/1000]	68.2%	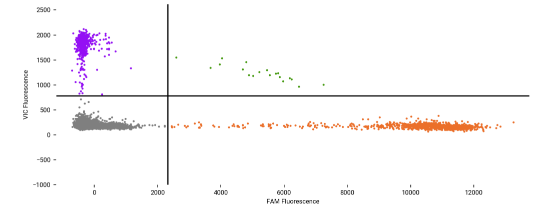
51 months Day 26 after transplantation	0.8%	0% [0/20]	NA	0.04%	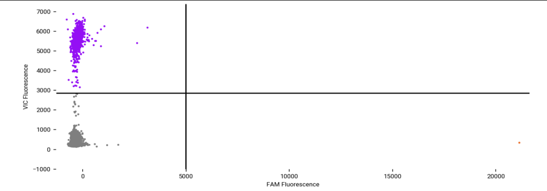
52 months Day 38 after transplantation	0.2%	0% [0/20]	NA	0.2%	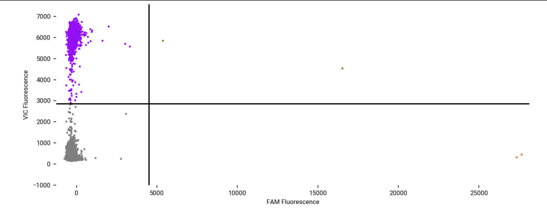

*Digital polymerase chain reaction (PCR) plots show variant-positive microwells (orange) and wild-type microwells (purple).
Abbreviations: FISH = fluorescence *in situ* hybridization, NA = not assessed, VAF = variant allele frequency.

Supplementary Table 3. *UBA1* mutation burdens in biopsy specimens at the referral.

Tissue	Histopathological findings	Digital PCR results for *UBA1* p.Met41Val variant	
		VAF	Analysis images*
Bone marrow	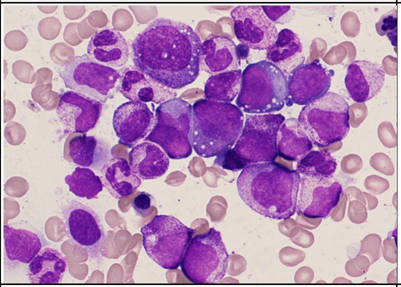	87.6%	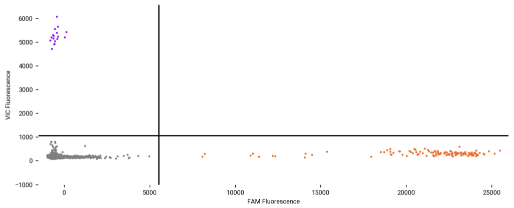
Skin	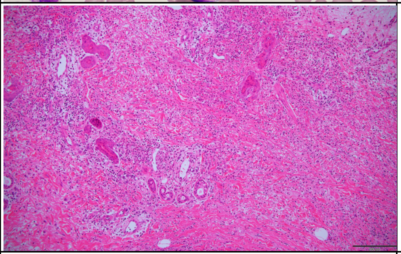	82.8%	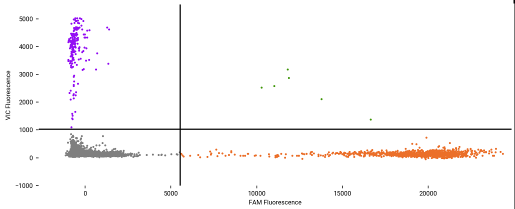
Ileum/ colon	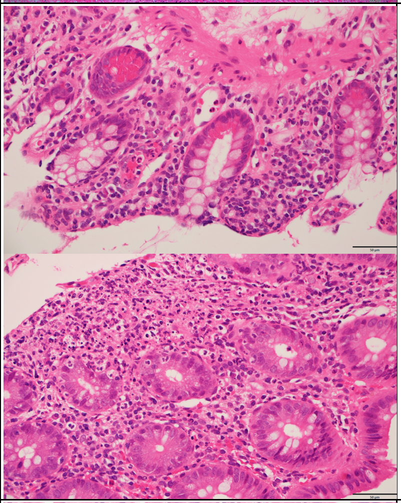	21.1%	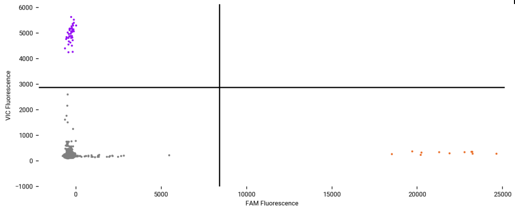
Stomach	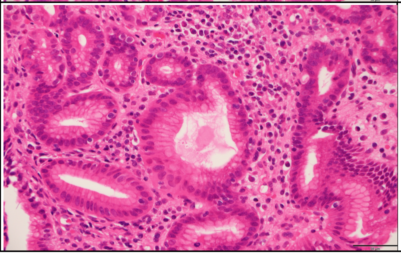	6.5%	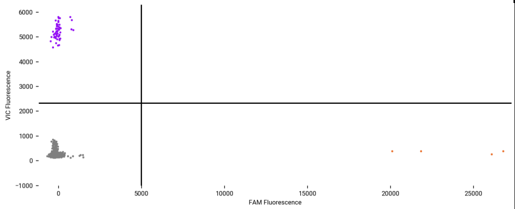

*Digital polymerase chain reaction (PCR) plots show variant-positive microwells (orange) and wild-type microwells (purple).
Abbreviations: VAF = variant allele frequency.

Supplementary Table 4. VEXAS syndrome with trisomy 8.

Reference and ID	Present case	Zaimoku et al.[4] Case 1	Zaimoku et al.[4] Case 3	Maeda et al.[5] RP97	Comont et al.[6] Patient 6	Comont et al.[6] Patient 11	Diarra et al.[7] UPN6	Gurnari et al.[8] UPN15	Ren et al.[9] P4
Age at onset	61	48	70	NA	53	63	50	55	70
UBA1 mutation	p.Met41Val	p.Met41Val	p.Met41Thr	p.Met41Val	p.Met41Val	p.Met41Val	p.Met41Val	p.Met41Thr	p.Met41Val
MDS phenotype	MDS-IB2	MDS-IB1	MDS-LB	MDS-IB1	MDS-IB1	MDS-IB1	MDS-IB1	MDS-LB	MDS-IB1
Additional mutation	*TET2* p. P1123fs (VAF 3.2%), *PHF6* p.K241E (VAF 4.7%)	NA	None	NA	NA	None	None	None	*TET2* p.E294* (VAF 2.0%)
Digestive symptom	Nausea, abdominal pain, diarrhea	NA	None	NA	Abdominal pain	Abdominal pain, diarrhea	None	None	None
Treatment	GC, CsA, AZA, CBT	ICx, UR-BMT	GC, UR-BMT	NA	GC, MMF, AZA	GC, AZA	GC, MMF, colchicine, AZA, UR-PBSCT	DMARDs, AZA, UR-PBSCT	NA
Outcome	Alive (2.5 years after CBT)	Died (5 months after BMT)	Died (28 months after BMT)	NA	Alive	Alive	Died (4 months after PBSCT)	Died (after PBSCT)	NA

Abbreviations: AZA = azacitidine, BMT = bone marrow transplantation, CBT = cord blood transplantation, CsA = cyclosporine, DMARDs = disease-modifying antirheumatic drugs, ICx = induction chemotherapy, GC = glucocorticoid, MDS = myelodysplastic syndrome, MDS-IB1 = MDS with increased blasts-1, MDS-IB2 = MDS with increased blasts-2, MDS-LB = MDS with low blasts, MMF = mycophenolate mofetil, NA = not assessed, PBSCT = peripheral blood stem cell transplantation, UR = unrelated donor.

Supplementary References

1. Yoshizato T, Nannya Y, Atsuta Y, et al. Genetic abnormalities in myelodysplasia and secondary acute myeloid leukemia: impact on outcome of stem cell transplantation. *Blood*. 2017;129(17):2347-2358.
2. Nannya Y, Tobiasson M, Sato S, et al. Postazacitidine clone size predicts long-term outcome of patients with myelodysplastic syndromes and related myeloid neoplasms. *Blood Adv*. 2023;7(14):3624-3636.
3. Horak P, Griffith M, Danos AM, et al. Standards for the classification of pathogenicity of somatic variants in cancer (oncogenicity): Joint recommendations of Clinical Genome Resource (ClinGen), Cancer Genomics Consortium (CGC), and Variant Interpretation for Cancer Consortium (VICC). *Genet Med*. 2022;24(5):986-998.
4. Zaimoku Y, Imi T, Hatada T, et al. Prevalence and outcome of VEXAS syndrome in unrelated hematopoietic cell transplantation for bone marrow failure. *Clin Exp Med*. 2025;25(1):300.
5. Maeda A, Tsuchida N, Uchiyama Y, et al. Efficient detection of somatic UBA1 variants and clinical scoring system predicting patients with variants in VEXAS syndrome. *Rheumatology (Oxford)*. 2024;63(8):2056-2064.
6. Comont T, Heiblig M, Rivière E, et al. Azacitidine for patients with Vacuoles, E1 Enzyme, X-linked, Autoinflammatory, Somatic syndrome (VEXAS) and myelodysplastic syndrome: data from the French VEXAS registry. *Br J Haematol*. 2022;196(4):969-974.
7. Diarra A, Duployez N, Fournier E, et al. Successful allogeneic hematopoietic stem cell transplantation in patients with VEXAS syndrome: a 2-center experience. *Blood Adv*. 2022;6(3):998-1003.
8. Gurnari C, Koster L, Baaij L, et al. Allogeneic hematopoietic cell transplantation for VEXAS syndrome: results of a multicenter study of the EBMT. *Blood Adv*. 2024;8(6):1444-1448.
9. Ren X, Wang M, Huo J, et al. The profile of clinical and laboratory features of Chinese VEXAS syndrome patients with hematological abnormalities: a single-center case series. *Front Immunol*. 2026;17:1794633.
